# Antibiotic Prescription Patterns for Acute Respiratory Infections in Rural Primary Healthcare Settings in Guangdong, China: Analysis of 162,742 Outpatient Prescriptions

**DOI:** 10.3390/antibiotics12020297

**Published:** 2023-02-01

**Authors:** Jiong Wang, Feifeng Li, Zhixu Chen, Yingyi Guo, Ningjing Liu, Baomo Liu, Shunian Xiao, Likang Yao, Jiahui Li, Chuyue Zhuo, Nanhao He, Guanyang Zou, Chao Zhuo

**Affiliations:** 1 State Key Laboratory of Respiratory Disease, The First Affiliated Hospital of Guangzhou Medical University, Guangzhou 510000, China; 2 Department of Respiratory and Critical Care Medicine, Meizhou People’s Hospital, Meizhou 514000, China; 3 Department of Respiratory and Critical Care Medicine, The First Affiliated Hospital of Sun Yat-sen University, Guangzhou 510000, China; 4 School of Public Health and Management, Guangzhou University of Chinese Medicine, Guangzhou 510000, China

**Keywords:** antibiotic prescriptions, acute respiratory infections, primary healthcare, rural area, antimicrobial resistance, antimicrobial stewardship, China

## Abstract

Overuse and inappropriate use of antibiotics are important contributors to bacterial antimicrobial resistance (AMR), especially in ambulatory primary healthcare (PHC) settings in low- and middle-income countries. This study aimed to investigate antibiotic prescription patterns among patients with acute respiratory infections (ARIs) in rural PHC facilities in the Guangdong Province, China. A total of 444,979 outpatient prescriptions were extracted from the electronic medical record system of 35 township health centers (THCs) and 2 community health centers (CHCs) between November 2017 and October 2018. We used the chi-square test to analyze the antibiotic prescription patterns and binary logistic regression to explore patient-related factors associated with antibiotic prescriptions. Of the 162,742 ARI prescriptions, 85.57% (*n* = 139,259) included at least one antibiotic. Among the 139,259 prescriptions with antibiotics, 37.82% (*n* = 52,666) included two or more antibiotics, 55.29% (*n* = 76,993) included parenteral antibiotics, and 56.62% (*n* = 78,852) included Watch group antibiotics. The binary logistic regression indicated that (1) female patients were slightly less likely to be prescribed antibiotics than males (adjusted odds ratio (OR) = 0.954, 95% confidence interval [CI] [0.928–0.981]; *p* = 0.001); and (2) compared to patients aged ≤5 years, those who were 6–15 years old (adjusted OR = 1.907, 95% CI [1.840–1.978]; *p* < 0.001), 16–60 years old (adjusted OR = 1.849, 95% CI [1.785–1.916]; *p* < 0.001), and >60 years old (adjusted OR = 1.915, 95% CI [1.810–2.026]; *p* < 0.001) were more likely to be prescribed antibiotics. The overuse and irrational use of antibiotics in PHC settings remain major healthcare challenges in rural Guangdong. Thus, it is imperative to implement targeted antimicrobial stewardship (AMS) policies to address this problem.

## 1. Introduction

Antimicrobial resistance (AMR) is an increasingly serious problem that threatens global public health [1]. A previous study estimated 4.95 million deaths related to drug-resistant bacterial infections worldwide in 2019, and among them, 1.27 million deaths were directly attributable to bacterial AMR [2]. The World Bank estimates that global economic losses will exceed USD 1 trillion per year, even under an optimistic low-impact AMR scenario, after 2030 [3]. However, other reports indicate that financial losses could increase to USD 3.5 billion per year over the next 30 years in Europe, North America, and Australia alone [4].

Overuse and irrational use of antibiotics are major drivers fueling the development of bacterial AMR [1], especially in primary healthcare (PHC) outpatient settings in low- and middle-income countries [5]. These issues have worsened during the COVID-19 pandemic, increasing improper antimicrobial exposure [6].

Antibiotic consumption is usually expressed in defined daily doses (DDDs). In China, 4.2 billion DDDs were consumed in 2015 [7], placing the country as the second largest consumer of antibiotics globally and setting a significant challenge [7,8]. To control AMR, in 2011, the Chinese Ministry of Health (MOH) launched the largest public health campaign, the National Antimicrobial Stewardship Program, which limited antibiotic prescriptions to less than 60% for admitted patients and 20% for outpatients [9]. In 2012, the Chinese MOH issued additional stringent administrative regulations for the clinical use of antimicrobials [9]. These new policies have been effective in large hospitals (secondary and tertiary hospitals) [8,10], but not so much in PHC locations [8,11,12,13]. Financial incentives from medical institutions, patient expectations and requests, and an unbalanced development among different regions and healthcare institutions are possible factors that may explain this disparity in healthcare settings [8,14,15].

In China, the PHC system has substantially helped reduce the burden of diseases, performing 53% of outpatient (4.1 billion visits) and 16% of inpatient (37.1 million hospital admissions) care in 2020 [16]. Therefore, it is essential to understand antibiotic prescription patterns in PHC settings. However, few studies have reported the latest findings on this matter [12,17,18,19], indicating a lack of evidence to support the development of antimicrobial stewardship (AMS) interventions.

Acute respiratory infections (ARIs) are commonly observed in PHC facilities [20]. Patients with upper respiratory tract infections (URTIs) often receive irrational treatment using antibiotics in this context [20,21]. However, most URTIs are of viral origin and self-limiting; consequently, antibiotics cannot reduce their duration or prevent complications [22]. The same happens in lower respiratory tract infections (LRTIs), such as acute bronchitis, where 44.5–93.5% of patients in China receive antibiotics despite commonly being of viral origin [23,24]. This study describes antibiotic prescription patterns for ARIs in rural township health centers (THCs) and community health centers (CHCs) in Guangdong, China. We aim to provide an evidence base to support the development of AMS in rural PHC institutions.

## 2. Methods

### 2.1. Data Extraction and Filtering

Even though Guangdong is one of the wealthiest provinces in China, it includes Shaoguan City, which is located in the north and considered one of the poorest areas. In rural Shaoguan, much of the younger population has temporarily moved to cities for employment, leaving behind children and elderly relatives. Our study covered two rural counties in Shaoguan, Lechang and Nanxiong. In 2018, Lechang County had 419,500 permanent residents, with an annual gross domestic product (GDP) of USD 4503 per person [25], and Nanxiong County had 337,500 permanent residents, with an annual GDP of USD 5203 per person [26].

We extracted 444,979 outpatient prescriptions from the electronic medical record system of 35 THCs and 2 CHCs in these two counties between November 2017 and October 2018. The electronic medical records contained basic patient information: name, medical card number, sex, age, disease diagnosis (coded according to the International Statistical Classification of Diseases 10th Revision (ICD-10)), chronic diseases, drug regimen, and prescription cost. The ARI diagnoses in our study included URTIs, coded by ICD-10 as J00 = acute nasopharyngitis (common cold), J01 = acute sinusitis, J02 = acute pharyngitis, J03 = acute tonsillitis, J04 = acute laryngitis and tracheitis, J05 = acute obstructive laryngitis (croup) and epiglottitis, and J06 = multiple acute upper respiratory tract infections of unknown sites; and the LRTI, J20 = acute bronchitis [27]. The chronic diseases included in our study were hypertension and diabetes.

**Figure 1:**
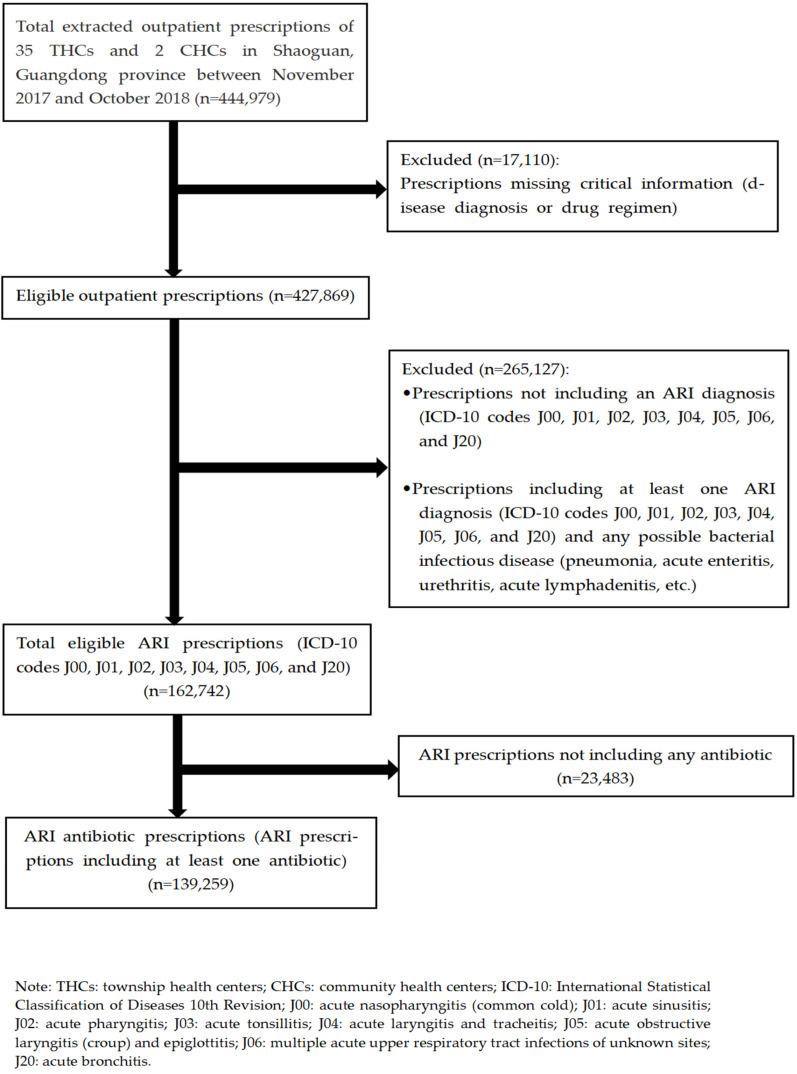
Flow chart of the selected acute respiratory infection (ARI) prescriptions for our study in rural Guangdong, China (November 2017 to October 2018).

Microsoft Excel (version 2019) was used to filter electronic medical records. First, prescriptions with missing critical information (e.g., disease diagnosis or drug regimen) and without an ARI diagnosis (ICD-10 codes J00, J01, J02, J03, J04, J05, J06, and J20) were excluded. Next, among all prescriptions containing at least one ARI diagnosis, we further excluded those presenting other possible bacterial infections (such as pneumonia, acute enteritis, urethritis, and acute lymphadenitis), as it was essential to ensure that antibiotics were prescribed only for ARIs. Lastly, in the selected ARI prescriptions, we screened those containing at least one antibiotic for systemic use and defined them as “ARI antibiotic prescriptions” (Figure 1). 

We classified the antibiotics used in our study according to the 2022 Anatomical Therapeutic Chemical (ATC) classification system recommended by the World Health Organization (WHO) [28]. Considering ATC code J01 (i.e., antibacterials for systemic use), we defined fourteen groups: ATC J01AA (tetracyclines), J01BA (amphenicols), J01CA (extended-spectrum penicillins), J01CE (beta-lactamase-sensitive penicillins), J01CR (penicillins/beta-lactamase inhibitors), J01DB (first-generation cephalosporins), J01DC (second-generation cephalosporins), J01DD (third-generation cephalosporins), J01EE (sulfonamides and trimethoprim), J01FA (macrolides), J01FF (lincosamides), J01GB (other aminoglycosides), J01MA (fluoroquinolones), and J01XD (imidazole derivatives). Additionally, antibiotics were also classified based on the 2021 WHO “Access, Watch and Reserve” (AWaRe) classification [29].

### 2.2. Key Indicators

Antibiotic prescription patterns were evaluated using primary and secondary outcome indicators. The primary outcome indicator was the rate of ARI antibiotic prescriptions. The secondary outcome indicators were expressed as the proportion of ARI antibiotic prescriptions, including parenteral antibiotics (intravenous (IV) or intramuscular (IM) administration), oral antibiotics, multiple antibiotics, AWaRe group of antibiotics, ATC group of antibiotics, and antiviral drugs. 

These key indicators were calculated as follows:

Antibiotic prescription rate = number of ARI prescriptions including at least one antibiotic for systemic use/total number of enrolled ARI prescriptions ×100%.

Parenteral antibiotic proportion = number of ARI prescriptions including at least one parenteral antibiotic/total number of ARI antibiotic prescriptions ×100%.

Oral antibiotic proportion = number of ARI prescriptions in which all included antibiotics were oral antibiotics/total number of ARI antibiotic prescriptions ×100%.

Multiple antibiotic proportion = number of ARI prescriptions including two or more individual antibiotic agents/total number of ARI antibiotic prescriptions ×100%.

Access group antibiotic proportion = number of ARI prescriptions in which all included antibiotics were Access group antibiotics/total number of ARI antibiotic prescriptions × 100%.

Watch group antibiotic proportion = number of ARI prescriptions including at least one antibiotic of the Watch group but without any antibiotic of the Reserve group or Not recommended group/total number of ARI antibiotic prescriptions × 100%.

Reserve group antibiotic proportion = number of ARI prescriptions including at least one antibiotic of the Reserve group but without any antibiotic of the Not recommended group/total number of ARI antibiotic prescriptions × 100%.

Not recommended group antibiotic proportion = number of ARI prescriptions including at least one antibiotic of the Not recommended group/total number of ARI antibiotic prescriptions × 100%.

Proportion of each ATC group of antibiotics = number of ARI prescriptions including at least one antibiotic from one ATC group/total number of ARI antibiotic prescriptions ×100%.

Proportion of antibiotics combined with antiviral drugs = number of ARI antibiotic prescriptions including at least one antiviral drug/total number of ARI antibiotic prescriptions ×100%.

### 2.3. Statistical Analysis

The statistical analysis was performed using the SPSS statistics software (version 25, IBM). We analyzed the overall antibiotic prescription patterns and stratified ARI prescriptions by sex (male or female), age (≤5, 6–15, 16–60, >60 years), and presence or absence of chronic diseases. We calculated the median prescription cost and interquartile range (IQR). We used the chi-square test to compare differences in antibiotic prescription patterns in the defined population groups and binary logistic regression (Enter method) to explore possible patient-related factors associated with antibiotic prescriptions. The covariates included patients’ age, sex, hypertension and diabetes status. Statistical significance was set at *p* < 0.05.

## 3. Results

### 3.1. General Characteristics of Patients Presenting ARIs

A total of 162,742 ARI prescriptions were included in the analysis; 52.65% (*n* = 85,685) belonged to male patients and 47.35% (*n* = 77,057) to females. The majority (89.47%, *n* = 145,600) of patients in this sample were ≤60 years old, with the highest proportion (34.13%, *n* = 55,539) of patients being aged ≤5 years, and with only 10.53% (*n* = 17,137) being >60 years old. Moreover, 95.51% (*n* = 155,430) lacked chronic diseases, and only 4.49% (*n* = 7312) were diagnosed with hypertension and/or diabetes. The most commonly identified diagnoses of ARIs were multiple acute upper respiratory tract infections of unknown sites (ICD-10 code J06) (68.10%, *n* = 110,825), followed by acute bronchitis (ICD-10 code J20) (16.37%, *n* = 26,648), acute pharyngitis (ICD-10 code J02) (9.50%, *n* = 15,455), and acute tonsillitis (ICD-10 code J03) (6.82%, *n* = 11,100) (Table 1).

### 3.2. Overall Patterns of the Antibiotic Prescriptions for ARIs

Most patients presenting ARIs (85.57%, *n* = 139,259) were prescribed at least one antibiotic, with 37.82% (*n* = 52,666) of them including multiple antibiotics. Moreover, 55.29% (*n* = 76,993) comprised parenteral antibiotics. According to the AWaRe classification, Watch group antibiotics accounted for the highest proportion (56.62%, *n* = 78,852), followed by the Access group (40.77%, *n* = 56,769) and the Not recommended group (2.61%, *n* = 3638), while no Reserve group antibiotics (0.00%, *n* = 0) were prescribed (Table 2). 

The most commonly prescribed antibiotics considering all ARI diagnoses in the sample were third-generation cephalosporins (29.17%, *n* = 40,618), first-generation cephalosporins (24.32%, *n* = 33,872), macrolides (20.33%, *n* = 28,306), and extended-spectrum penicillins (18.91%, *n* = 26,327) (Table 2). In relation to the different ARI diagnoses, third-generation cephalosporins were the most frequently prescribed antibiotics for ICD-10 codes J01 (acute sinusitis), J02 (acute pharyngitis), J03 (acute tonsillitis), J04 (acute laryngitis and tracheitis), J06 (multiple acute upper respiratory tract infections of unknown sites), and J20 (acute bronchitis). As an exception, macrolides were prescribed most frequently for ICD-10 code J00 (acute nasopharyngitis; common cold) (63.51%, *n* = 235) (Table S1). Regarding individual agents within each antibiotic group, cefalexin (15.43%, *n* = 21,493) was the most common antibiotic for all ARIs in the sample, followed by amoxicillin (14.55%, *n* = 20,264), cefixime (12.74%, *n* = 17,739), and ceftriaxone (11.62%, *n* = 16,179). When evaluating the prescribed individual antibiotic agent per ARI diagnosis, we found that erythromycin was the most frequent for ICD-10 code J00 (55.41%), levofloxacin and metronidazole for J01 (17.27%), amoxicillin for J02 (14.80%), amoxicillin–clavulanic acid for J03 (22.34%), cefixime for J04 (24.55%), cefalexin for J06 (17.62%), and azithromycin for J20 (15.70%) (Table S2).

Furthermore, 45.86% (*n* = 63,861) of the antibiotic prescriptions included at least one antiviral drug. Based on the median prescription cost, the average ARI antibiotic prescription cost was higher than that of ARI prescriptions without antibiotics (USD 3.60 vs. USD 2.17) (Table 2).

### 3.3. Patterns Stratified by Sex

There were no statistically significant differences between males and females in the antibiotic prescription rate, parenteral, oral, and multiple antibiotic proportions. The antibiotic prescription rate for male and female patients exceeded 85% (*p* = 0.373). Despite the sex, more than half of the patients were prescribed parenteral antibiotics (*p* = 0.089), and approximately 38% were prescribed multiple antibiotics (*p* = 0.901). However, male patients were prescribed a higher proportion of antibiotics in the Watch group (57.18% vs. 56.00%; *p* < 0.001) and antibiotics combined with antiviral drugs (47.05% vs. 44.54%; *p* < 0.001) than female patients (Table 3). Similar results were obtained regarding second- and third-generation cephalosporins, and penicillins/beta-lactamase inhibitor prescriptions, which were prescribed at a higher proportion in males than females (9.57% vs. 8.86%, 30.19% vs. 28.03%, 12.88% vs. 10.70%, respectively; *p* < 0.001) (Table S3).

### 3.4. Patterns Stratified by Age

All referenced age groups showed antibiotic prescription rates higher than 80% and even beyond 88% in the three groups aged >5 years (80.24% ≤5 years vs. 88.56% 6–15 years, 88.13% 16–60 years, and 88.29% >60 years; *p* < 0.001). Additionally, general practitioners (GPs) tended to prescribe parenteral antibiotics (40.87% ≤5 years vs. 55.84% 6–15 years, 62.50% 16–60 years, and 76.82% >60 years; *p* < 0.001) and multiple antibiotics (26.17% ≤5 years vs. 40.17% 6–15 years, 43.37% 16–60 years, and 51.06% >60 years; *p* < 0.001) according to the patient’s increasing age. Moreover, patients aged ≤5 years received a higher proportion of antibiotics from the Watch group (59.99% ≤5 years vs. 56.90% 6–15 years, 53.15% 16–60 years, and 55.40% >60 years; *p* < 0.001) and antibiotics combined with antiviral drugs (52.26% ≤5 years vs. 48.02% 6–15 years, 38.50% 16–60 years, and 41.45% >60 years; *p* < 0.001) when compared to other age groups (Table 4). The most commonly prescribed antibiotics in patients aged ≤5 and 6–15 years were third-generation cephalosporins (33.59% and 28.35%, respectively), while first-generation cephalosporins were the most frequent prescriptions in patients aged 16–60 (30.13%) and >60 years (31.20%). Fluoroquinolones were barely used in patients aged ≤5 (0.00%) and 6–15 years (0.04%) compared to those aged 16–60 (8.09%) and >60 years (8.21%) (*p* < 0.001) (Table S4).

### 3.5. Patterns Stratified by Chronic Disease

Regarding patients with or without chronic diseases, the antibiotic prescription rate exceeded 85% in both groups (87.57% vs. 85.48%; *p* < 0.001), the proportion of Watch group antibiotics was beyond 56% (56.63% vs. 56.62%; *p* < 0.001), and the proportion of antibiotics combined with antiviral drugs was more than 41% (41.87 vs. 46.05; *p* < 0.001). The groups showed significant differences in the route of administration and the number of antibiotics prescribed. Patients with chronic diseases received a higher proportion of parenteral antibiotics (77.49% vs. 54.22%; *p* < 0.001) and multiple antibiotics (51.98% vs. 37.14%; *p* < 0.001) than those without chronic diseases (Table 5). Moreover, patients with chronic diseases were more likely to receive treatment with first- and third- generation cephalosporins, extended-spectrum penicillins, lincosamides, and fluoroquinolones (*p* < 0.001), but showed a lower proportion of prescriptions for macrolides and penicillins/beta-lactamase inhibitors (*p* < 0.001) (Table S5).

### 3.6. Patient-Related Factors Associated with Antibiotic Prescriptions

After using binary logistic regression, we found that GPs were slightly less inclined to prescribe antibiotics in female patients than in male patients (adjusted odds ratio (OR) = 0.954, 95% confidence interval [CI] [0.928–0.981]; *p* = 0.001). As evidenced before, we confirmed that patients aged >5 years were more likely to be prescribed antibiotics, particularly those aged >60 years (adjusted OR=1.915, 95% CI [1.810–2.026]; *p* < 0.001). Moreover, patients with hypertension appeared less prone to receiving antibiotics than those without hypertension (adjusted OR = 0.894, 95% CI [0.822–0.973]; *p* = 0.009). However, there was no significant difference in antibiotic prescriptions between patients with and without diabetes (adjusted OR = 1.129, 95% CI [0.966–1.319]; *p* = 0.128) (Table 6).

## 4. Discussion

Our study demonstrates that antibiotic overuse and improper use in patients presenting ARIs are prevalent in rural PHC facilities in Guangdong, China. The antibiotic prescription rate was excessively high, as well as the proportions of multiple antibiotics, parenteral, and Watch group antibiotics. Moreover, we found that patients’ sex and age were key factors associated with GPs’ prescription behaviors in the studied PHC facilities.

### 4.1. Overall Patterns of the Antibiotic Prescriptions for ARIs

We found that 85.57% of the total ARI prescriptions in the eligible sample included at least one antibiotic, in line with a previous report from a systematic review in China, where 91.1% of outpatients with URTIs were prescribed antibiotics in PHC settings [21]. However, these findings showed a significantly higher antibiotic prescription rate than that expected by the Chinese MOH of less than 20% for outpatients [9] and the average level of antibiotic use recommended by the WHO (less than 30%) [30], as well as the average antibiotic prescription rate for URTI outpatients in secondary and tertiary hospitals in 28 provinces in China (40.8%) [31].

Among all ARI antibiotic prescriptions in our study, 37.82% included multiple antibiotics, a slightly lower figure than previously reported for URTI outpatients in Chinese PHC settings (55.5%) [21], but significantly higher compared to secondary and tertiary hospitals in the same systematic review (15.6% and 16.7%, respectively) [21] and an additional study (11.6%) in twenty-eight Chinese provinces [31].

We also found that more than half of the ARI antibiotic prescriptions (55.29%) included parenteral antibiotics, a much higher rate than that recommended by the WHO (less than 20%) [30] and those employed in European outpatient settings (2.04%) [32]. Antibiotic overuse via IV and IM administration is concerning, as parenteral antibiotics could expose patients to additional potential side effects and safety issues when compared to oral antibiotics [30,33]. However, since many Chinese patients believe that drugs administered via IV and IM injections work faster and more efficiently than oral drugs, there is pressure on physicians to prescribe them [34]. In addition, IV and IM administrations can bring greater financial benefits to physicians in China than oral medications [34,35].

Our study identified that Watch group antibiotics were more frequently prescribed for ARIs than those in the Access group (56.62% vs. 40.77%), in accordance with previous reports on children under 18 years old with respiratory tract infections in outpatient PHC settings in Beijing, China (89.0% vs. 10.5%) [36]. The WHO encourages a priority use for Access group antibiotics and recommends that at least 60% of the overall antibiotic use at the country level should belong to this group [37]. Since antibiotics in the Watch group are generally broad-spectrum, they are more likely to induce bacterial AMR compared to Access group antibiotics (generally narrow-spectrum antibiotics) [38]. Broad-spectrum antibiotics can potentially drive AMR via multiple non-causative bacteria exposed to antibiotic selection pressure as well as causing microbiota dysbiosis by modification of the normal human flora [39,40]. Thus, intensive use of broad-spectrum antibiotics often results in human superinfections, especially drug-resistant superinfections [41].

The observed patterns in our study notably deviate from the Chinese national guidelines, according to which ARIs are mainly viral and should not be treated with antibiotics unless accompanied by evidence of bacterial infection [42,43]. Furthermore, the guidelines discourage multiple-antibiotics treatment, except for specific conditions (e.g., severe bacterial infections), and recommend oral antibiotics over IV or IM injections [44]. In addition, GPs are encouraged to prioritize targeted narrow-spectrum antibiotics [44].

### 4.2. Patterns Stratified by Sex, Age, and Chronic Disease

In our study, female patients with ARIs were less likely to be prescribed antibiotics than male patients. Zhao et al. found similar results in a larger-scale study of patients with URTIs in ambulatory and emergency departments of secondary and tertiary hospitals in 28 provinces of China [31].

Regarding age, our results indicate that patients were more likely to be prescribed parenteral antibiotics as they became older, consistent with the findings in rural Anhui, China [13]. In addition, the prescription of multiple antibiotics increased with age, in line with the fact that multidrug therapy is more common in the elderly [45]. Interestingly, a previous study in rural areas of western China also reported consistent results [46]. Patients that were ≤5 years of age showed a lower antibiotic prescription rate (80.24%) and decreased use of parenteral antibiotics (40.87%) and multiple antibiotics (26.17%) compared to other age groups in the sample. However, these proportions were much higher than those in patients younger than 5 years with acute URTIs in urban areas of China (27.1%, 27.0%, and 5.7%, respectively) [47]. Additionally, fluoroquinolones were barely used in patients aged ≤15 years in the sample, which is potentially related to national guideline recommendations by which these antibiotics should not be prescribed to children under 18 years of age due to possible adverse effects on bone development [44].

Patients with ARIs presenting chronic diseases were prescribed parenteral and multiple antibiotics at a higher proportion. This is also related to the fact that most of these patients (66.89%, 4283/6403) were over 60 years of age in our study, suggesting a strong link between this age group, the route of administration, and prescription of multiple antibiotics, as previously stated. Moreover, it is worth mentioning that the low prevalence of chronic diseases (4.49%) in our study might be related to the fact that most (60.89%) patients presenting ARIs were ≤15 years old.

### 4.3. Reasons for Overuse and Irrational Use of Antibiotics

Despite the implementation of China’s zero-markup drug policy to eliminate profit from medicine sales, financial incentives may still exist for medical institutions [8]. Before the policy was established in 2009, Chinese medical institutions heavily relied on drug sales to generate income and support the recovery of costs (up to 50% of total revenue was generated from drug sales) [9]. Although the government provided an additional subsidy to replace the removal of 15% of the drug profit margin, PHC institutions still found it challenging to compensate for this financial imbalance [48]. In particular, even though drugs would still be sold at their original cost, an additional fee could be implemented when administering IV or IM injections [48,49].

Irrational antibiotic prescriptions can also be attributed to diagnostic uncertainty [20,49,50]. In our study, 45.86% of ARI antibiotic prescriptions also included antiviral drugs. When GPs are unable to identify whether the infection is caused by bacteria or virus, they will likely prescribe a combination of antibacterial and antiviral drugs, as they believe that either of these two kinds of drugs (or even both) will attack the pathogen(s) causing the infection [49]. Moreover, GPs’ uncertainty in identifying a specific type of pathogenic bacteria could lead to a prescription of broad-spectrum antibiotics and multiple antibiotics, which are believed to tackle a wider range of pathogenic bacteria [49]. The strategies implemented to confront these diagnostic challenges reflect GPs’ intention to quickly improve their patient’s health and gain a good professional reputation [34,49]. However, accurate diagnosis can be obstructed by the lack of proper equipment, laboratory support, and point-of-care testing (POCT) in rural PHC institutions [49,50].

Another important factor may be the lack of GPs’ professional knowledge and training on antibiotic use, leading to improper use of antibiotics [17,18]. In 2018, only 29% of licensed physicians in THCs received university medical education, this being significantly lower for those in secondary and tertiary hospitals (76%) [51]. Furthermore, continued education in PHC institutions is insufficient [48]. Even though the Chinese MOH requires GPs in this location to take annual training courses and earn target credits [52], a study revealed that more than a third did not comply in 2016 [48].

In addition, patient expectations and demands may influence irrational antibiotic prescriptions [14]. Patients in rural areas had lower education levels with little understanding of antibiotic use and AMR (indeed, they often considered antibiotics as “anti-inflammatories”) and most likely push GPs to prescribe antibiotics without weighing the consequences [20,49]. For fear of losing patients, and in the context of deteriorating doctor–patient relationships, GPs tend to satisfy their patients by prescribing more antibiotics, including parenteral, broad-spectrum, and even multiple antibiotics to “speed up” patients’ recovery [34,49]. Particularly, for most families in traditional Chinese culture, protecting their children’s health is the highest priority [53]. Especially in the studied rural areas, where only grandparents and their grandchildren are left at home, GPs may relent to the caregivers’ pressure to prescribe antibiotics [54], as also seen in our study for children ≤5 years.

### 4.4. Policy Implications and Recommendations

As evidenced, urgent actions should be adopted to control the overuse and inappropriate use of antibiotics for ARIs and improve the situation in rural PHC institutions in Guangdong, China.

First, the government should increase funding for rural PHC institutions to reduce financial dependence on drug sales, and strengthen diagnostic equipment, especially for POCT services. POCT, as a diagnostic test performed near or at the site of patient care, provides faster test results than traditional laboratory testing [55]. Particularly, C-reactive protein POCT can rapidly and efficiently assist physicians in the identification of bacterial infections, reducing the diagnostic uncertainty and unnecessary antibiotic prescriptions for ARIs [20,50,56].

Second, improving antibiotic use awareness among GPs and patients is imperative. Examples of measures include strengthening GPs’ professional training, encouraging the study of clinical guidelines, and implementing prescription peer review [57,58]. Specific curricula and education for medical students should also be developed to shape attitudes and behaviors at their career start [8,59]. Increasing public education (e.g., through mass media) and doctor–patient communications regarding the rational use of antibiotics and AMR control (especially on the severe outcomes of AMR) [8,58], would be essential to improve awareness.

Third, the government should strengthen the supervision of antibiotic prescriptions in PHC institutions. A study in Shenzhen, China, indicated that strict enforcement of AMS interventions (measures such as setting antibiotic management objectives, assigning management responsibilities, and developing reward and punishment mechanisms) implemented by local health authorities could make substantial and constant reductions in antibiotic prescriptions in PHC settings [60]. Moreover, tight surveillance on antibiotic utilization and bacterial AMR in PHC settings should be implemented so that tailored AMS actions may be applied, especially during emergencies, such as the COVID-19 pandemic. Although national surveillance systems have been frequently conducted in China since 2005 for secondary and tertiary hospitals, they have not yet been established for PHC settings [8].

Finally, simple metrics for antibiotic prescriptions should be developed that are easily understood by GPs and policymakers, for the purpose of assessing and improving the quality of antibiotic prescriptions more accurately. The proportion of each WHO AWaRe antibiotic group, the percentage of parenteral administration, and the ratio of broad-to-narrow-spectrum antibiotics comprise good examples to follow [61,62,63]. However, it is worth mentioning that new and targeted indicators should be developed based on the local prevalence of pathogenic bacteria, AMR status, and antibiotic prescription patterns.

### 4.5. Strengths and Limitations

Our study covered 35 THCs and 2 CHCs in rural areas, with a large sample size (*n* = 162,742), providing strong evidence of irrational antibiotic prescription practices. Moreover, this analysis contributes to implementing targeted AMS policies in rural PHC facilities. Nonetheless, it presents some apparent limitations. First, the antibiotic prescription rate of outpatient ARI prescriptions may have been slightly biased after excluding prescriptions with missing diagnosis and/or drug regimen. However, they accounted for only 3.85% (17,110/444,979) of the total number extracted.

Second, caution should be taken when making generalizations and interpretations of our findings. Despite the large sample size of enrolled prescriptions, our study investigated only two counties in Guangdong Province, China; therefore, results can only be extrapolated to similar populations. Moreover, other potential confounding variables, such as GPs’ characteristics and more patient chronic diseases (apart from hypertension and diabetes), which may also affect the decision to prescribe antibiotics, were not analyzed in the selected ARI prescriptions. Future studies should include more cities and provinces and control more confounding variables to improve the generalizability of the findings.

Third, quality indicators of antibiotic use required for AMS implementation, for example, adherence to guidelines, could not be analyzed in our study due to insufficient information in the electronic medical record system in rural PHC settings (such as information on patient symptoms, signs, and medical test result). However, since antibiotics are generally not required for ARIs, we have been able to demonstrate the overuse and irrational use of antibiotics in a general sense.

Finally, our study did not include antibiotic prescriptions from village clinics and community health stations (PHC facilities are one level lower than THCs and CHCs), fields that should also be explored for a more comprehensive understanding of antibiotic prescription practices in rural PHC settings in China.

## 5. Conclusions

Irrational antibiotic prescription for ARIs is a serious concern in rural PHC facilities in Guangdong, China. GPs often overprescribe antibiotics, including parenteral antibiotics, Watch group antibiotics, and multiple combinations of them. We found that patients’ sex and age are critical factors associated with this issue. Targeted AMS interventions are required to improve rational prescription practices in these settings. Potential implementations should be focused on, but not be limited to, enhancing funding, diagnostic equipment, healthcare provider and patient education, and antimicrobial surveillance, as well as implementing the WHO AWaRe’s metrics, and developing feasible quality indicators adapted to the local context.

## Figures and Tables

**Table 1:** General characteristics of patients with acute respiratory infections (ARIs) in the eligible prescription sample.

	**Number of Prescriptions, *n* (%)**
**Total ARI prescriptions**	*n* = 162,742
**Sex**	
Male	85,685 (52.65%)
Female	77,057 (47.35%)
**Age group (in years) ^a^**	
≤5	55,539 (34.13%)
6–15	43,543 (26.76%)
16–60	46,518 (28.58%)
>60	17,137 (10.53%)
**Without any chronic disease**	155,430 (95.51%)
**With at least one chronic disease ^b^**	7312 (4.49%)
With hypertension	6422 (3.95%)
With diabetes	1740 (1.07%)
**ARI diagnosis (ICD-10 codes) ^c^**	
J00	438 (0.27%)
J01	121 (0.07%)
J02	15,455 (9.50%)
J03	11,100 (6.82%)
J04	1309 (0.80%)
J05	0 (0.00%)
J06	110,825 (68.10%)
J20	26,648 (16.37%)

Note: ^a^ There were five patients (0.00%) whose ages were unknown. ^b^ A total of 850 (0.52%) patients had more than one chronic disease. ICD-10: International Statistical Classification of Diseases 10th Revision. ^c^ A total of 3151 (1.94%) prescriptions included more than one ARI diagnosis category. J00: acute nasopharyngitis (common cold); J01: acute sinusitis; J02: acute pharyngitis; J03: acute tonsillitis; J04: acute laryngitis and tracheitis; J05: acute obstructive laryngitis (croup) and epiglottitis; J06: multiple acute upper respiratory tract infections of unknown sites; J20: acute bronchitis.

**Table 2:** Overall antibiotic prescription patterns for patients with ARIs in the eligible prescription sample.

	**Number of Prescriptions, *n* (%)**
** Total ARI prescriptions**	*n* = 162,742
** Antibiotic prescription rate**	139,259 (85.57%)
** Administration route**	
Parenteral antibiotic	76,993 (55.29%)
Oral antibiotic	62,266 (44.71%)
** Number of antibiotics**	
1	86,593 (62.18%)
2	42,581 (30.58%)
≥3	10,085 (7.24%)
** Antibiotic AWaRe group**	
Access	56,769 (40.77%)
Watch	78,852 (56.62%)
Reserve	0 (0.00%)
Not recommended	3638 (2.61%)
** Antibiotic ATC group ^a^**	
J01DD (third-generation cephalosporins)	40,618 (29.17%)
J01DB (first-generation cephalosporins)	33,872 (24.32%)
J01FA (macrolides)	28,306 (20.33%)
J01CA (extended-spectrum penicillins)	26,327 (18.91%)
J01CR (penicillins/beta-lactamase inhibitors)	16,500 (11.85%)
J01DC (second-generation cephalosporins)	12,858 (9.23%)
J01GB (other aminoglycosides)	10,920 (7.84%)
J01FF (lincosamides)	10,803 (7.76%)
J01XD (imidazole derivatives)	8068 (5.79%)
J01CE (beta-lactamase-sensitive penicillins)	5395 (3.87%)
J01MA (fluoroquinolones)	4573 (3.28%)
J01EE (sulfonamides and trimethoprim)	57 (0.04%)
J01AA (tetracyclines)	13 (0.01%)
J01BA (amphenicols)	5 (0.00%)
** Antibiotics combined with antiviral drugs**	63,861 (45.86%)
** Median prescription cost (IQR) (USD)**	
Median cost for all ARI prescriptions	3.38 (1.99–5.63)
Median cost for ARI antibiotic prescriptions	3.60 (2.18–5.94)
Median cost for ARI prescriptions without antibiotics	2.17 (0.97–3.72)

Note: ARIs: acute respiratory infections; AWaRe: “Access, Watch and Reserve” classification; ATC: Anatomical Therapeutic Chemical classification system. ^a^ Antibiotic ATC group patterns for each ARI diagnosis category and individual antibiotic agent patterns for ARIs are presented in Table S1 and Table S2 (Supplementary Materials), respectively. IQR: interquartile range.

**Table 3:** Antibiotic prescription patterns stratified by sex ^a^.

	**Number of Prescriptions, *n* (%)**		
	**Male**	**Female**	***p* Value**
** ARI prescriptions**	85,685 (52.65%)	77,057 (47.35%)	
** Antibiotic prescription rate**	73,258 (85.50%)	66,001 (85.65%)	0.373
** Administration route**			0.089
Parenteral antibiotic	40,660 (55.50%)	36,333 (55.05%)	
Oral antibiotic	32,598 (44.50%)	29,668 (44.95%)	
** Multiple antibiotics**	27,694 (37.80%)	24,972 (37.84%)	0.901
** Antibiotic AWaRe group**			<0.001
Access	29,231 (39.90%)	27,538 (41.72%)	
Watch	41,890 (57.18%)	36,962 (56.00%)	
Reserve	0 (0.00%)	0 (0.00%)	
Not recommended	2137 (2.92%)	1501 (2.27%)	
** Antibiotics combined with antiviral drugs**	34,466 (47.05%)	29,395 (44.54%)	<0.001

Note: ^a^ Antibiotic ATC group patterns stratified by sex are shown in Table S3 (Supplementary Materials). ARI: acute respiratory infection; AWaRe: “Access, Watch and Reserve” classification.

**Table 4:** Antibiotic prescription patterns stratified by age (in years) ^a^.

	**Number of Prescriptions, *n* (%)**				
	**≤5**	**6–15**	**16–60**	**>60**	***p* Value**
** ARI prescriptions**	55,539 (34.13%)	43,543 (26.76%)	46,518 (28.58%)	17,137 (10.53%)	
** Antibiotic prescription rate**	44,564 (80.24%)	38,562 (88.56%)	40,998 (88.13%)	15,130 (88.29%)	<0.001
** Administration route**					<0.001
Parenteral antibiotic	18,212 (40.87%)	21,532 (55.84%)	25,623 (62.50%)	11,623 (76.82%)	
Oral antibiotic	26,352 (59.13%)	17,030 (44.16%)	15,375 (37.50%)	3507 (23.18%)	
** Multiple antibiotics**	11,664 (26.17%)	15,492 (40.17%)	17,781 (43.37%)	7726 (51.06%)	<0.001
** Antibiotic AWaRe group**					<0.001
Access	16,327 (36.64%)	15,299 (39.67%)	18,616 (45.41%)	6524 (43.12%)	
Watch	26,736 (59.99%)	21,940 (56.90%)	21,792 (53.15%)	8382 (55.40%)	
Reserve	0 (0.00%)	0 (0.00%)	0 (0.00%)	0 (0.00%)	
Not recommended	1501 (3.37%)	1323 (3.43%)	590 (1.44%)	224 (1.48%)	
** Antibiotics combined with antiviral drugs**	23,288 (52.26%)	18,516 (48.02%)	15,783 (38.50%)	6272 (41.45%)	<0.001

Note: ^a^ Five prescriptions were excluded due to missing age data. Antibiotic ATC group patterns stratified by age are presented in Table S4 (Supplementary Materials). ARI: acute respiratory infection; AWaRe: “Access, Watch and Reserve” classification.

**Table 5:** Antibiotic prescription patterns stratified by chronic disease ^a^.

	**Number of Prescriptions, *n* (%)**		
	**With Chronic Diseases**	**Without Chronic Diseases**	***p* Value**
** ARI prescriptions**	7312 (4.49%)	155,430 (95.51%)	
** Antibiotic prescription rate**	6403 (87.57%)	132,856 (85.48%)	<0.001
** Administration route**			<0.001
Parenteral antibiotic	4962 (77.49%)	72,031 (54.22%)	
Oral antibiotic	1441 (22.51%)	60,825 (45.78%)	
** Multiple antibiotics**	3328 (51.98%)	49,338 (37.14%)	<0.001
** Antibiotic AWaRe group**			<0.001
Access	2677 (41.81%)	54,092 (40.71%)	
Watch	3626 (56.63%)	75,226 (56.62%)	
Reserve	0 (0.00%)	0 (0.00%)	
Not recommended	100 (1.56%)	3538 (2.66%)	
** Antibiotics combined with antiviral drugs**	2681 (41.87%)	61,180 (46.05%)	<0.001

Note: ^a^ Antibiotic ATC group patterns stratified by chronic disease are shown in Table S5 (Supplementary Materials). ARI: acute respiratory infection; AWaRe: “Access, Watch and Reserve” classification.

**Table 6:** Patient-related factors associated with antibiotic prescriptions using binary logistic regression.

	**Adjusted OR**	**95% CI**	***p* Value**
**Sex: Ref = Male**			
Female	0.954	[0.928, 0.981]	0.001
**Chronic disease: Ref = Without hypertension**			
With hypertension	0.894	[0.822, 0.973]	0.009
**Chronic disease: Ref = Without diabetes**			
With diabetes	1.129	[0.966, 1.319]	0.128
**Age (in years): Ref = ≤5**			
6–15	1.907	[1.840, 1.978]	<0.001
16–60	1.849	[1.785, 1.916]	<0.001
>60	1.915	[1.810, 2.026]	<0.001

Note: OR: odds ratio; CI: confidence interval; Ref: reference group.

## Data Availability

The data supporting the study findings were obtained from the local health authorities in Shaoguan, Guangdong, China. However, restrictions apply to the availability of these data since they were used under license for the current study and are not publicly available. However, data are available from the authors upon reasonable request and with permission from the health authorities of Shaoguan.

## Supplementary Materials

The following supporting information can be downloaded at: https://www.mdpi.com/article/10.3390/antibiotics12020297/s1. Table S1. Antibiotic Anatomical Therapeutic Chemical (ATC) group patterns for each acute respiratory infection (ARI) diagnosis category in the eligible prescription sample. Table S2. Individual antibiotic agent patterns for patients with ARIs in the eligible prescription sample. Table S3. Antibiotic ATC group patterns stratified by sex. Table S4. Antibiotic ATC group patterns stratified by age (in years). Table S5. Antibiotic ATC group patterns stratified by chronic disease.
