# Supplementary material for: Antibiotic Prescription Patterns for Acute Respiratory Infections in Rural Primary Healthcare Settings in Guangdong, China: Analysis of 162,742 Outpatient Prescriptions
Source: Antibiotics (Basel). 2023 Feb 1;12(2):297. doi: 10.3390/antibiotics12020297 (PMC9952100; doi:10.3390/antibiotics12020297)
Supplement: Supplementary file 1 [file antibiotics-12-00297-s001.zip › antibiotics-1833182-supplementary.pdf]

Table S1. Antibiotic Anatomical Therapeutic Chemical (ATC) group patterns for each acute respiratory infection (ARI) diagnosis category in the eligible prescription sample [a].

	ARI Diagnosis (ICD-10 Codes)						
	**J00** [b]	**J01** [b]	**J02** [b]	**J03** [b]	**J04** [b]	**J06** [b]	**J20** [b]
**Antibiotic prescription rate**	370 (84.47%)	110 (90.91%)	13,707 (88.69%)	10,235 (92.21%)	1157 (88.39%)	92,724 (83.67%)	23,926 (89.79%)
**Antibiotic ATC group**							
J01DD (third-generation cephalosporins)	138 (37.30%)	29 (26.36%)	4097 (29.89%)	3191 (31.18%)	414 (35.78%)	26,760 (28.86%)	7018 (29.33%)
J01DB (first-generation cephalosporins)	26 (7.03%)	21 (19.09%)	2954 (21.55%)	1912 (18.68%)	270 (23.34%)	25,751 (27.77%)	3089 (12.91%)
J01FA (macrolides)	235 (63.51%)	11 (10.00%)	2614 (19.07%)	1224 (11.96%)	210 (18.15%)	17,779 (19.17%)	6858 (28.66%)
J01CA (extended-spectrum penicillins)	61 (16.49%)	18 (16.36%)	2835 (20.68%)	2128 (20.79%)	160 (13.83%)	16,397 (17.68%)	4996 (20.88%)
J01CR (penicillins/beta-lactamase inhibitors)	36 (9.73%)	10 (9.09%)	2088 (15.23%)	2877 (28.11%)	199 (17.20%)	8238 (8.88%)	3703 (15.48%)
J01DC (second-generation cephalosporins)	4 (1.08%)	14 (12.73%)	852 (6.22%)	816 (7.97%)	34 (2.94%)	9066 (9.78%)	2780 (11.62%)
J01GB (other aminoglycosides)	17 (4.59%)	0 (0.00%)	1704 (12.43%)	402 (3.93%)	90 (7.78%)	6929 (7.47%)	1924 (8.04%)
J01FF (lincosamides)	21 (5.68%)	18 (16.36%)	956 (6.97%)	927 (9.06%)	50 (4.32%)	7637 (8.24%)	1383 (5.78%)
J01XD (imidazole derivatives)	38 (10.27%)	21 (19.09%)	1893 (13.81%)	1364 (13.33%)	38 (3.28%)	4386 (4.73%)	406 (1.70%)
J01CE (beta-lactamase-sensitive penicillins)	2 (0.54%)	3 (2.73%)	438 (3.20%)	354 (3.46%)	13 (1.12%)	4267 (4.60%)	567 (2.37%)
J01MA (fluoroquinolones)	3 (0.81%)	19 (17.27%)	538 (3.93%)	116 (1.13%)	23 (1.99%)	3083 (3.32%)	821 (3.43%)
J01EE (sulfonamides and trimethoprim)	0 (0.00%)	0 (0.00%)	6 (0.04%)	4 (0.04%)	0 (0.00%)	41 (0.04%)	6 (0.03%)
J01AA (tetracyclines)	0 (0.00%)	0 (0.00%)	0 (0.00%)	3 (0.03%)	0 (0.00%)	6 (0.01%)	4 (0.02%)
J01BA (amphenicols)	0 (0.00%)	0 (0.00%)	0 (0.00%)	0 (0.00%)	0 (0.00%)	5 (0.01%)	0 (0.00%)

Note: [a] No patients (n = 0) were diagnosed with acute obstructive laryngitis (croup) and epiglottitis (ICD-10 code J05) in the eligible ARI prescription sample. ICD-10: International Statistical Classification of Diseases 10th Revision. ICD-10 code J00: acute nasopharyngitis (common cold); J01: acute sinusitis; J02: acute pharyngitis; J03: acute tonsillitis; J04: acute laryngitis and tracheitis; J06: multiple acute upper respiratory tract infections of unknown sites; J20: acute bronchitis. [b] For each ARI diagnosis category, the sum of proportions of all fourteen antibiotic ATC groups was greater than 100% since prescriptions included more than one antibiotic ATC group.

Table S2. Individual antibiotic agent patterns for patients with ARIs in the eligible prescription sample [a].

	Total ARI Prescriptions [b,c]	ARI Diagnosis (ICD-10 Codes)						
		**J00** [b,c]	**J01** [b,c]	**J02** [b,c]	**J03** [b,c]	**J04** [b,c]	**J06** [b,c]	**J20** [b,c]
Cefixime	17,739 (12.74%)	7 (1.89%)	7 (6.36%)	1979 (14.44%)	2172 (21.22%)	284 (24.55%)	10,410 (11.23%)	3138 (13.12%)
Ceftriaxone	16,179 (11.62%)	129 (34.86%)	17 (15.45%)	1675 (12.22%)	817 (7.98%)	111 (9.59%)	10,959 (11.82%)	2540 (10.62%)
Cefotaxime	3006 (2.16%)	0 (0.00%)	7 (6.36%)	95 (0.69%)	151 (1.48%)	10 (0.86%)	2539 (2.74%)	225 (0.94%)
Ceftazidime	3001 (2.15%)	3 (0.81%)	0 (0.00%)	550 (4.01%)	94 (0.92%)	30 (2.59%)	1975 (2.13%)	361 (1.51%)
Ceftizoxime	2466 (1.77%)	0 (0.00%)	0 (0.00%)	84 (0.61%)	81 (0.79%)	5 (0.43%)	2017 (2.18%)	953 (3.98%)
Cefotaxime-sulbactam	25 (0.02%)	0 (0.00%)	0 (0.00%)	4 (0.03%)	4 (0.04%)	0 (0.00%)	13 (0.01%)	4 (0.02%)
Cefoperazone-sulbactam	19 (0.01%)	0 (0.00%)	0 (0.00%)	3 (0.02%)	0 (0.00%)	17 (1.47%)	0 (0.00%)	0 (0.00%)
Cefalexin	21,493 (15.43%)	7 (1.89%)	16 (14.55%)	1879 (13.71%)	1010 (9.87%)	70 (6.05%)	16,338 (17.62%)	2232 (9.33%)
Cefradine	12,056 (8.66%)	19 (5.14%)	3 (2.73%)	985 (7.19%)	839 (8.20%)	193 (16.68%)	9280 (10.01%)	829 (3.46%)
Cefazolin	500 (0.36%)	0 (0.00%)	2 (1.82%)	109 (0.80%)	82 (0.80%)	15 (1.30%)	258 (0.28%)	34 (0.14%)
Erythromycin	15,113 (10.85%)	205 (55.41%)	3 (2.73%)	1733 (12.64%)	606 (5.92%)	42 (3.63%)	10,072 (10.86%)	2710 (11.33%)
Azithromycin	9524 (6.84%)	4 (1.08%)	2 (1.82%)	344 (2.51%)	497 (4.86%)	130 (11.24%)	4993 (5.38%)	3757 (15.70%)
Roxithromycin	2695 (1.94%)	1 (0.27%)	3 (2.73%)	348 (2.54%)	99 (0.97%)	35 (3.03%)	1848 (1.99%)	508 (2.12%)
Midecamycin	811 (0.58%)	25 (6.76%)	3 (2.73%)	170 (1.24%)	18 (0.18%)	2 (0.17%)	406 (0.44%)	188 (0.79%)
Dirithromycin	747 (0.54%)	0 (0.00%)	0 (0.00%)	8 (0.06%)	6 (0.06%)	1 (0.09%)	691 (0.75%)	43 (0.18%)
Clarithromycin	115 (0.08%)	0 (0.00%)	1 (0.91%)	19 (0.14%)	7 (0.07%)	7 (0.61%)	74 (0.08%)	8 (0.03%)
Amoxicillin	20,264 (14.55%)	57 (15.41%)	14 (12.73%)	2029 (14.80%)	1678 (16.39%)	96 (8.30%)	12,891 (13.90%)	3739 (15.63%)
Ampicillin	6915 (4.97%)	8 (2.16%)	5 (4.55%)	949 (6.92%)	492 (4.81%)	41 (3.54%)	3959 (4.27%)	1502 (6.28%)
Mezlocillin	826 (0.59%)	0 (0.00%)	0 (0.00%)	80 (0.58%)	58 (0.57%)	32 (2.77%)	544 (0.59%)	112 (0.47%)
Piperacillin	6 (0.00%)	0 (0.00%)	0 (0.00%)	0 (0.00%)	0 (0.00%)	0 (0.00%)	6 (0.01%)	0 (0.00%)
Amoxicillin-clavulanic acid	13,027 (9.35%)	36 (9.73%)	9 (8.18%)	1694 (12.36%)	2286 (22.34%)	184 (15.90%)	6785 (7.32%)	2622 (10.96%)
Piperacillin-sulbactam	3594 (2.58%)	0 (0.00%)	1 (0.91%)	388 (2.83%)	547 (5.34%)	16 (1.38%)	1607 (1.73%)	1102 (4.61%)

Piperacillin-tazobactam	172 (0.12%)	0 (0.00%)	0 (0.00%)	21 (0.15%)	59 (0.58%)	0 (0.00%)	63 (0.07%)	29 (0.12%)
Cefuroxime	7815 (5.61%)	3 (0.81%)	13 (11.82%)	742 (5.41%)	588 (5.74%)	28 (2.42%)	4993 (5.38%)	1521 (6.36%)
Cefaclor	2344 (1.68%)	0 (0.00%)	1 (0.91%)	36 (0.26%)	165 (1.61%)	0 (0.00%)	1740 (1.88%)	437 (1.83%)
Cefprozil	2002 (1.44%)	1 (0.27%)	0 (0.00%)	35 (0.26%)	37 (0.36%)	0 (0.00%)	1740 (1.88%)	680 (2.84%)
Cefmetazole	679 (0.49%)	0 (0.00%)	0 (0.00%)	39 (0.28%)	27 (0.26%)	1 (0.09%)	528 (0.57%)	217 (0.91%)
Cefoxitin	167 (0.12%)	0 (0.00%)	0 (0.00%)	9 (0.07%)	21 (0.21%)	0 (0.00%)	131 (0.14%)	6 (0.03%)
Cefminox	122 (0.09%)	0 (0.00%)	0 (0.00%)	4 (0.03%)	11 (0.11%)	5 (0.43%)	102 (0.11%)	0 (0.00%)
Gentamicin	10,573 (7.59%)	10 (2.70%)	0 (0.00%)	1683 (12.28%)	397 (3.88%)	87 (7.52%)	6660 (7.18%)	1872 (7.82%)
Amikacin	349 (0.25%)	1 (0.27%)	0 (0.00%)	21 (0.15%)	6 (0.06%)	3 (0.26%)	270 (0.29%)	52 (0.22%)
Clindamycin	10,779 (7.74%)	21 (5.68%)	18 (16.36%)	956 (6.97%)	926 (9.05%)	50 (4.32%)	7615 (8.21%)	1382 (5.78%)
Lincomycin	24 (0.02%)	0 (0.00%)	0 (0.00%)	0 (0.00%)	1 (0.01%)	0 (0.00%)	22 (0.02%)	1 (0.00%)
Metronidazole	7933 (5.7%)	38 (10.27%)	19 (17.27%)	1852 (13.51%)	1331 (13.00%)	37 (3.20%)	4335 (4.68%)	389 (1.63%)
Ornidazole	117 (0.08%)	0 (0.00%)	2 (1.82%)	34 (0.25%)	30 (0.29%)	0 (0.00%)	49 (0.05%)	12 (0.05%)
Tinidazole	18 (0.01%)	0 (0.00%)	0 (0.00%)	7 (0.05%)	3 (0.03%)	1 (0.09%)	2 (0.00%)	5 (0.02%)
Benzylpenicillin	5393 (3.87%)	2 (0.54%)	3 (2.73%)	438 (3.20%)	354 (3.46%)	13 (1.12%)	4266 (4.60%)	566 (2.37%)
Benzathine benzylpenicillin	2 (0.00%)	0 (0.00%)	0 (0.00%)	0 (0.00%)	0 (0.00%)	0 (0.00%)	1 (0.00%)	1 (0.00%)
Levofloxacin	3736 (2.68%)	3 (0.81%)	19 (17.27%)	468 (3.41%)	97 (0.95%)	18 (1.56%)	2380 (2.57%)	779 (3.26%)
Norfloxacin	618 (0.44%)	0 (0.00%)	0 (0.00%)	65 (0.47%)	22 (0.21%)	4 (0.35%)	489 (0.53%)	41 (0.17%)
Ciprofloxacin	301 (0.22%)	0 (0.00%)	0 (0.00%)	8 (0.06%)	0 (0.00%)	1 (0.09%)	291 (0.31%)	1 (0.00%)
Sulfamethoxazole and trimethoprim	57 (0.04%)	0 (0.00%)	0 (0.00%)	6 (0.04%)	4 (0.04%)	0 (0.00%)	41 (0.04%)	6 (0.03%)
Doxycycline	13 (0.01%)	0 (0.00%)	0 (0.00%)	0 (0.00%)	3 (0.03%)	0 (0.00%)	6 (0.01%)	4 (0.02%)
Chloramphenicol	5 (0.00%)	0 (0.00%)	0 (0.00%)	0 (0.00%)	0 (0.00%)	0 (0.00%)	5 (0.01%)	0 (0.00%)

Note: ARIs: acute respiratory infections. [a] No patients ($n = 0$) were diagnosed with acute obstructive laryngitis (croup) and epiglottitis (ICD-10 code J05) in the eligible ARI prescription sample. ICD-10: International Statistical Classification of Diseases 10th Revision. [b] Proportion of each individual antibiotic agent = number of ARI prescriptions including the individual antibiotic agent/number of ARI antibiotic prescriptions ×100%. [c] The sum of proportions of all forty-four individual antibiotic agents was greater than 100% since

prescriptions included more than one individual antibiotic agent. ICD-10 code J00: acute nasopharyngitis (common cold); J01: acute sinusitis; J02: acute pharyngitis; J03: acute tonsillitis; J04: acute laryngitis and tracheitis; J06: multiple acute upper respiratory tract infections of unknown sites; J20: acute bronchitis.

Table S3. Antibiotic ATC group patterns stratified by sex.

	Number of Prescriptions, *n* (%)		
	**Male**	**Female**	***p* Value**
**Antibiotic ATC group**			
J01DD (third-generation cephalosporins)	22,117 (30.19%)	18,501 (28.03%)	<0.001
J01DB (first-generation cephalosporins)	17,278 (23.59%)	16,594 (25.14%)	<0.001
J01FA (macrolides)	14,696 (20.06%)	13,610 (20.62%)	0.009
J01CA (extended-spectrum penicillins)	13,165 (17.97%)	13,162 (19.94%)	<0.001
J01CR (penicillins/beta-lactamase inhibitors)	9438 (12.88%)	7062 (10.70%)	<0.001
J01DC (second-generation cephalosporins)	7012 (9.57%)	5846 (8.86%)	<0.001
J01GB (other aminoglycosides)	5587 (7.63%)	5333 (8.08%)	0.002
J01FF (lincosamides)	5442 (7.43%)	5361 (8.12%)	<0.001
J01XD (imidazole derivatives)	4314 (5.89%)	3754 (5.69%)	0.109
J01CE (beta-lactamase-sensitive penicillins)	2890 (3.94%)	2505 (3.80%)	0.149
J01MA (fluoroquinolones)	2030 (2.77%)	2543 (3.85%)	<0.001
J01EE (sulfonamides and trimethoprim)	26 (0.04%)	31 (0.05%)	0.290
J01AA (tetracyclines)	4 (0.01%)	9 (0.01%)	0.164
J01BA (amphenicols)	1 (0.00%)	4 (0.01%)	0.197

Note: ATC: Anatomical Therapeutic Chemical classification system.

Table S4. Antibiotic ATC group patterns stratified by age (in years) [a].

	Number of Prescriptions, *n* (%)				
	**≤5**	**6–15**	**16–60**	**>60**	***p* Value**
**Antibiotic ATC group**					
J01DD (third-generation cephalosporins)	14,967 (33.59%)	10,933 (28.35%)	10,087 (24.60%)	4630 (30.60%)	<0.001
J01DB (first-generation cephalosporins)	7533 (16.90%)	9266 (24.03%)	12,352 (30.13%)	4720 (31.20%)	<0.001
J01FA (macrolides)	9143 (20.52%)	9381 (24.33%)	7555 (18.43%)	2226 (14.71%)	<0.001
J01CA (extended-spectrum penicillins)	6269 (14.07%)	6319 (16.39%)	10,039 (24.49%)	3698 (24.44%)	<0.001
J01CR (penicillins/beta-lactamase inhibitors)	6201 (13.91%)	5827 (15.11%)	3413 (8.32%)	1059 (7.00%)	<0.001
J01DC (second-generation cephalosporins)	4844 (10.87%)	4118 (10.68%)	2834 (6.91%)	1062 (7.02%)	<0.001
J01GB (other aminoglycosides)	3646 (8.18%)	3332 (8.64%)	3016 (7.36%)	926 (6.12%)	<0.001
J01FF (lincosamides)	1930 (4.33%)	2702 (7.01%)	4060 (9.90%)	2109 (13.94%)	<0.001
J01XD (imidazole derivatives)	1224 (2.75%)	2550 (6.61%)	3293 (8.03%)	1000 (6.61%)	<0.001
J01CE (beta-lactamase-sensitive penicillins)	766 (1.72%)	1050 (2.72%)	2322 (5.66%)	1257 (8.31%)	<0.001
J01MA (fluoroquinolones)	1 (0.00%)	14 (0.04%)	3316 (8.09%)	1242 (8.21%)	<0.001
J01EE (sulfonamides and trimethoprim)	4 (0.01%)	13 (0.03%)	37 (0.09%)	3 (0.02%)	<0.001
J01AA (tetracyclines)	0 (0.00%)	1 (0.00%)	11 (0.03%)	1 (0.01%)	<0.001
J01BA (amphenicols)	0 (0.00%)	0 (0.00%)	4 (0.01%)	1 (0.01%)	0.052

Note: ATC: Anatomical Therapeutic Chemical classification system. [a] Five prescriptions were excluded due to missing age data.

Table S5. Antibiotic ATC group patterns stratified by chronic disease.

	Number of Prescriptions, *n* (%)		
	**With Chronic Diseases**	**Without Chronic Diseases**	***p* Value**
**Antibiotic ATC group**			
J01DD (third-generation cephalosporins)	2100 (32.80%)	38,518 (28.99%)	<0.001
J01DB (first-generation cephalosporins)	1910 (29.83%)	31,962 (24.06%)	<0.001
J01FA (macrolides)	924 (14.43%)	27,382 (20.61%)	<0.001
J01CA (extended-spectrum penicillins)	1597 (24.94%)	24,730 (18.61%)	<0.001
J01CR (penicillins/beta-lactamase inhibitors)	463 (7.23%)	16,037 (12.07%)	<0.001
J01DC (second-generation cephalosporins)	451 (7.04%)	12,407 (9.34%)	<0.001
J01GB (other aminoglycosides)	459 (7.17%)	10,461 (7.87%)	0.040
J01FF (lincosamides)	931 (14.54%)	9872 (7.43%)	<0.001
J01XD (imidazole derivatives)	504 (7.87%)	7564 (5.69%)	<0.001
J01CE (beta-lactamase-sensitive penicillins)	398 (6.22%)	4997 (3.76%)	<0.001
J01MA (fluoroquinolones)	509 (7.95%)	4064 (3.06%)	<0.001
J01EE (sulfonamides and trimethoprim)	0 (0.00%)	57 (0.04%)	0.115
J01AA (tetracyclines)	0 (0.00%)	13 (0.01%)	1.000
J01BA (amphenicols)	1 (0.02%)	4 (0.00%)	0.210

Note: ATC: Anatomical Therapeutic Chemical classification system.
